# The Generation and Characterization of Recombinant Protein and Antibodies of *Clostridium perfringens* Beta2 Toxin

**DOI:** 10.1155/2016/5708468

**Published:** 2016-09-08

**Authors:** Jin Zeng, Fuyang Song, Yi Yang, Chenjie Ma, Guangcun Deng, Yong Li, Yujiong Wang, Xiaoming Liu

**Affiliations:** ^1^Key Laboratory of Ministry of Education for Conservation and Utilization of Special Biological Resources in the Western China, Ningxia University, Yinchuan, Ningxia 750021, China; ^2^College of Life Science, Ningxia University, Yinchuan, Ningxia 750021, China; ^3^Center of Laboratory Medicine, The General Hospital of Ningxia Medical University, Yinchuan, Ningxia 750004, China

## Abstract

*Introduction. Clostridium perfringens* (*C. perfringens*) beta2 toxin (CPB2) is an important virulent factor of necrotic enteritis in both animals and humans. However, studies of its pathogenic roles and functional mechanisms have been hampered due to the difficulty of purification and lack of specific antibodies against this toxin.* Methods.* A recombinant His-tagged* C. perfringens* beta2 (rCPB2) toxin and monoclonal antibodies (McAbs) against CPB2 were generated and characterized by assays of cytotoxicity, immunoblotting, ELISA, neutralization, and immunofluorescence.* Results.* A His-tagged rCPB2 with integrity and cytotoxicity of native CPB2 was purified from* E. coli *expressing system, which exhibited a moderate cytotoxicity on NCM460 human intestinal epithelial cells. The rCPB2 could induce apoptotic cell death rather than necrotic death in part through a pathway involved in caspase-3 signaling. Mechanistically, rCPB2 was able to first bind to cell membrane and dynamically translocate into cytoplasm for its cytotoxic activity. Three McAbs 1E23, 2G7 and 2H7 were characterized to be able to immunologically react with CPB2 and neutralize rCPB2 cytotoxicity on NCM460 cells.* Conclusion.* These results indicated the rCPB2 and antibodies generated in this study are useful tools for studies of biological functions and pathogenic mechanisms of CPB2 in future, which warrants for further investigations.

## 1. Introduction

*Clostridium perfringens* (*C. perfringens*) is a ubiquitous bacterium broadly distributed in environment. It also presents as a normal intestinal flora of humans and domestic animals.* C. perfringens* is an arsenal producing a wide range of bacterial toxins, of which genes encoding 17 distinct toxins have been identified in the chromosome or plasmids in this bacterial species [1]. According to its capacity to produce four major types of alpha, beta, epsilon, and iota toxins, bacteria of this species could be further grouped into five different toxin types from A to E [1]. Historically, this species of bacteria was first found as a cause of food poisoning by McClung in mid-1940s, and it thus has been linked to gastrointestinal (GI) diseases in human [2].* C. perfringens* beta toxin (CPB, also known as CPB1) is a lethal toxin produced by* C. perfringens* type C and type B, which is a 35 kDa protein able to form pores on membranes of susceptible cells, leading to cell distension and lysis [3]. The 50% lethal dose of CPB was determined at 310 ng/kg when it was administered intravenously (i.v.) [3]. In addition to CPB1,* C. perfringens* was found to produce another beta toxin, the beta2 toxin (CPB2) with a molecular weight (MW) of ~28 kDa, which was first identified from a* C. perfringens* strain isolated from a piglet that died of necrotizing enterocolitis [4]. Intriguingly, the CPB2 has no significant homologies with other* Clostridium* toxins, and the mode of its action has not been elucidated yet [4]. Despite its encoding gene,* cpb2* was found on plasmids of* C. perfringens* isolates [5, 6]. Pathologically, both CPB1 and CPB2 are thought to be important virulent factors of the necrotic enteritis in humans and animals, particularly in piglets [4, 7, 8], and a presence of* cpb2*-positive* C*.* perfringens* strains in the intestine has been associated with intestinal diseases in humans [9], ruminants [10], horses [7], and piglets [11, 12].

It has been reported that over 97% of* C. perfringens* isolates from pig diarrhea could produce CPB2 [12], in addition to* C. perfringens* strains producing alpha toxin (CPA). Both CPA and CPB2 were found in horses suffering from typhlocolitis and other intestinal disorders, particularly those treated with the aminoglycoside antibiotic gentamicin [7]. Equally noteworthy, the presence of* cpb2* gene was also reported in* C. perfringens* isolated from humans [9, 13]. In a study conducted by Carman et al.,* C. perfringens* isolates carrying a* cpb2* gene were found to colonize in 8 of 43 (23%) healthy subjects [13]. In another study, Fisher et al. reported that the* cpb2* gene was detected in 75% of* C. perfringens* isolates from cases of antibiotic-associated diarrhea (AAD) and sporadic diarrhea, among which 97% of these isolates could produce CPB2* in vitro* [9]. These results provide conflicting evidence as to whether CPB2 is associated with human enteric diseases in clinical settings, although* C. perfringens* isolates bearing genes of* cpb2* and* C. perfringens* enterotoxin (CPE),* cpe,* have been linked to human antibiotic-associated/sporadic diarrhea (AAD/SD) [9]. In these clinical isolates, the production of CPB2 as an accessory toxin in human* cpe*-positive* C. perfringens* type A bacteria needs to be further confirmed [9, 14]. Similarly, Ronco et al. recently compared genomes of* C. perfringens* isolates from healthy and diseased poultry and pigs, and they found that all isolates from healthy (*n* = 4) and diseased (*n* = 6) chickens, healthy (*n* = 4) and diseased (*n* = 5) turkeys, and diseased pigs (*n* = 5) harbored the* cpb2* gene [15]. These studies clearly imply that CPB2 may be a virulent factor in enteric diseases [4, 11]. However, unlike* C. perfringens* alpha toxin (CPA), the pathogenic role of CPB2 in the pig enteric disease caused by an infection of* C. perfringens* type C isolates has not been characterized yet [4, 7, 8], and its cytopathological functions and mechanisms of cytotoxicity are also currently unknown, largely owing to a difficulty in the purification of CPB2 toxin and a lack of antibodies specifically against the toxin.

On a view of above studies, it is a necessity to have a purified CPB2 toxin and antibody to this toxin for further investigation of its biological functions and pathological mechanisms. In the present study, we reported characterizations of purified recombinant CPB2 toxin (rCPB2) and monoclonal antibodies (McAbs) against CPB2, by determining the integrity and cytotoxicity of rCPB2 and immunological reactions of McAbs employed in immunoblotting, immunofluorescent, ELISA, and neutralizing assays. The results suggest that the rCPB2 toxin expressed by* E. coli* has biological functions, and the characterized McAbs can be used for studies of cytotoxic mechanism and determination of CPB2 in clinical settings. These reagents thus will be useful tools for investigation of biological functionality and pathogenic mechanism of CPB2 in future.

## 2. Materials and Methods

### 2.1. Bacterial Strains, Cell Lines, Plasmids, Reagents, and Chemicals

All chemicals were of analytical grade and purchased from Sigma (St. Louis, MO, USA), unless stated otherwise. pMD18-T vector, DNA restriction enzymes,* Taq* DNA polymerase, and T4 DNA ligase were the products of NEB (New England Biolabs Inc. Ipswich, MA, USA). Kits for gel extraction and plasmid purification were products of TianQen Biological Inc. (Beijing, China).* Escherichia coli* BL21(DE3) cells were purchased from EMD Millipore (Billerica, MA, USA). pTIG-Trx plasmid was a gift of Dr. Professor Jinlin Wang from Academy of Military Medical Sciences [16].* Clostridium perfringens* China isolates C58-1 (type B) and C59-44 (type C) were purchased from the China Institute of Veterinary Drug Control (Beijing, China). The primers were synthesized by Shanghai Sangon Company (Shanghai, China). The intestinal epithelial cell line NCM460 was procured from the National Centre for Cell Science (Shanghai, China).

### 2.2. Construction of Plasmid Expressing rCPB2 in* E. coli*

**Figure 1:**
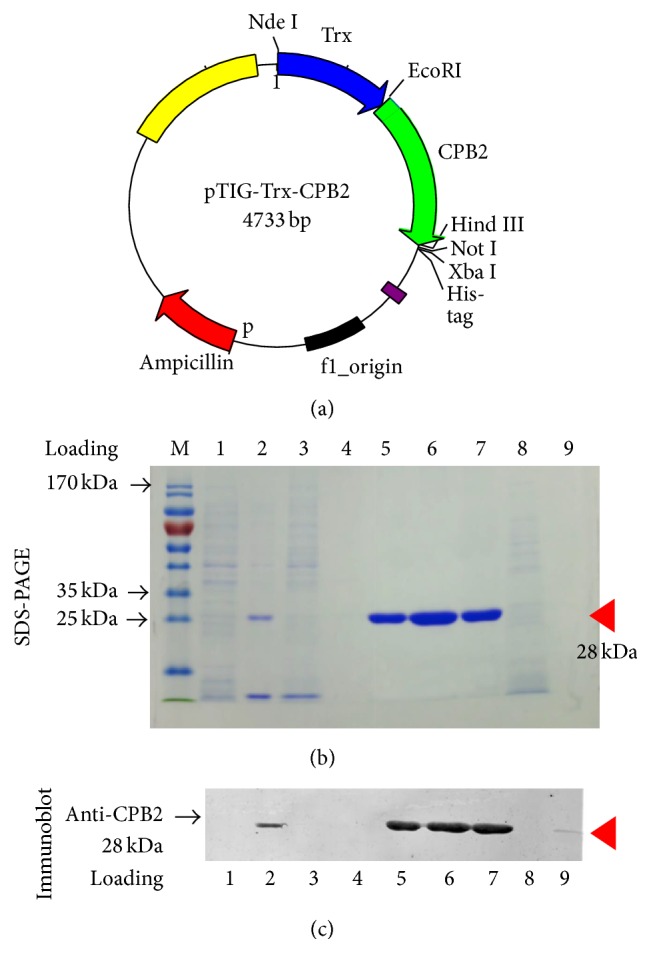
Purification and characterization of recombinant toxin beta2 of* Clostridium perfringens* type C (rCPB2). The expression of CPB2 was induced by IPTG in* E. coli* BL21 (DE3) cells harboring His-tagged CPB2 expressing plasmid pTIG-Trx-CPB2; the bacterial cells expressed rCPB2 lysed by sonication, and the soluble fraction of cells was used for purification of rCPB2 by HPLC AKTA PURIFIER PH/C10 system using a Ni-NTA column with a linear gradient elution of 0–1000 mM imidazole. (a) The map of pTIG-Trx-CPB2 plasmid capable of expressing rCPB2 in* E. coli* cells. (b) An image of the expression and purification of recombinant CPB by a Coomassie stained SDS-PAGE (12%) analysis.* Lane M*, a ladder of molecular weights (MW).* Lane 1*, a lysate of* E. coli* BL21(DE3) cells transformed with plasmid pTIG-Trx.* Lane 2*, a lysate of* E. coli* BL21(DE3) cells transformed with plasmid pTIG-Trx-CPB2 induced by IPTG.* Lane 3*, a fraction of HPLC elution at 10 mM of imidazole.* Lane 4*, a fraction of HPLC elution at 25 mM of imidazole.* Lanes 5–7*, fractions of HPLC elution at 75 mM of imidazole.* Lane 8*, cell lysate of C59-44.* Lane 9*, the culture supernatant of C59-44 (type C). A band of protein of interest with expected MW of ~28 kDa was observed in lanes 2 and 5–7 (arrowhead). (c) An immunoblotting analysis of the specificity and integrity of purified rCPB2 using monoclonal antibody against CPB2; a specific band was detected in purified fractions of rCPB2 (lanes 5–7); the lysate of* E. coli* harboring* cpb2* recombinant plasmid (lane 2) and supernatant of* C. perfringens* C59-44 strain (lane 9).

Bacterial genomic DNA from the* C. perfringens* type C strain China isolate C59-44 was isolated using a bacterial genomic DNA isolation kit (TianQen Biological Inc., China). Based on the* cpb2* DNA sequence deposited in GenBank (GenBank: AY609177.1), the following primer sets were designed and used for amplifying the encoding regions of the* cpb2* gene: CPB2-F: 5′Cgg*AATTC*TAATgAAAgAAATCgACgCTTAT3′ and CPB2-R: 5′GAAT*gCggCCgC*TgCACAATACCCTTCACC3′, and restriction sites of EcoRI and Not I were included in the 3′-ends of the primers, respectively. The PCR amplified DNA fragment was first cloned into pMD18-T vector for sequencing. The sequenced confirmed* cpb2* gene DNA was further in-frame subcloned into the upstream of His-tag of pTIG-Trx bacterial expression vector using appropriate restriction sites (EcoRI and NotI) [16]. The resultant plasmid was designated as pTIG-Trx-CPB2 (Figure 1(a)) and transformed into* E. coli* BL21(DE3) cells for induction of the expression of His-tagged CPB2 protein.

### 2.3. Induction of the Expression of Recombinant CPB2 Toxin (rCPB2)

The above pTIG-Trx-CPB2 plasmid was transformed into* E. coli* BL21 (DE3) competent cells and selected with Ampicillin (Amp) antibiotics. Single bacterial colonies were isolated and cultured in the LB medium containing 50 *μ*g/mL of Ampicillin in a 37°C incubator with shaking overnight. The cultured bacterial cells were used as seeds for induction of transgene expression. 1.0% (V/V) of the seed culture was inoculated into LB broth medium containing 50 *μ*g/mL of Ampicillin with a 37°C incubation to log phase (determined by optical density 600 nm [OD_600_ = 0.6]). IPTG (isopropyl *β*-D-1-thiogalactopyranoside) was added at a final concentration of 1 mM and the mixture was cultured for an additional 4 h. Following the IPTG induction, the bacterial cells were harvested by centrifugation and the bacterial pellet was resuspended in 10 pellet volumes of lysis buffer (20 mM Tris-Cl buffer, 50 mM NaCl, pH 8.5). The supernatants were analyzed for expression of the recombinant protein on 12% sodium dodecyl sulphate polyacrylamide gel electrophoresis (SDS-PAGE).

### 2.4. Purification of His-Tagged rCPB2 Toxin

Since the rCPB2 expressed in pTIG-Trx vector system was His-tagged, it could be purified using nickel-nitrilotriacetic acid (Ni-NTA) columns. Briefly, the IPTG-induced bacterial cells harboring pTIG-Trx-CPB2 plasmid were harvested by centrifugation at 10,000 ×g for 20 min at 4°C. The pellet was resuspended in 10 volumes of pellet of lysis buffer (20 mM Tris-HCl, 100 mM NaCl, pH 8.0) containing 10 mg/mL of lysozyme and incubated on ice for 2 hr. After incubation, the cell suspension was sonicated for 10 cycles (30 s each with cooling for 30 s between the cycles) (SONICS, USA) prior to the cell extraction, followed by centrifugation at 12000 ×g for 20 min at 4°C. The supernatant (10 mL) was purified using a Ni-NTA column (GE Healthcare, USA) by a gradient HPLC (AKTA PURIFIER PH/C10). Following the binding to column, the column was washed with lysis buffer containing 10 mM imidazole. Elution was performed by the use of buffer containing increasing concentrations of imidazole (10, 75, and 100 mM). The fractions were analyzed by 12% SDS-PAGE. Endotoxin of the purified rCPB2 proteins was removed using a Toxin Eraser TM Endotoxin Removal Kit (GenScript, USA) before use.

### 2.5. Generation of Hybridoma Cell Lines Producing Monoclonal Antibodies to CPB2

Ten potential epitopes of CPB2 protein were selected for the generation of murine hybridoma cell lines producing monoclonal antibodies (McAbs). These epitopes were predicted based on an in-house software developed by Abmart Inc. (Shanghai, China). Peptides of 12 amino acids (AAs) of each epitope were synthesized and consequently integrated into an Abmart antigen system to generate mixture of antigens for further immunization of mice and screening McAbs. Immunized mice were used for generation of hybridoma cell lines producing McAbs as described elsewhere [17]. Hybridoma cell lines were injected into Balb/C mice to produce ascites containing McAbs.

### 2.6. SDS-PAGE and Immunoblotting Assay

The protein concentration was determined by the Bradford method against known standards. The expression and purity of protein of interest were ascertained by resolving the protein on a 12% sodium dodecyl sulphate polyacrylamide gel (SDS–PAGE), followed by Coomassie G250 blue silver staining. The specificity and integrity of proteins were determined by an immunoblotting assay using mouse antibodies specifically against CPB2 toxin (self-generated by Abmart as mentioned in Section 2.5), followed by a secondary IRDye 800CW goat anti-mouse or IRDye 680CW goat anti-rabbit (LI-COR). Immune complexes were detected using the Odyssey dual-infrared fluorescence system. Antibodies against human beta-actin, caspase-3, and Bax were products of proteintech (Rosemont, IL, USA).

### 2.7. Cytotoxicity Assay

The activity of the rCPB2 proteins was evaluated using NCM460 cells essentially as previously described [18]. The rCPB2 toxin was diluted in RPMI-1640 Medium Modified supplemented with 10% fetal calf serum (FCS) and added to the cells followed by incubation at 37°C for 12 h. The control wells were treated with an equal volume of the medium only. The toxicity of rCPB2 was accessed by determining the cell viability of the NCM460 cells by a 3-(4,5-dimethylthiazol-2-yl)-2,5-diphenyltetrazolium bromide (MTT) assay as described everywhere [19]. The dose of rCPB2 that causes 50% cell death (cytotoxicity 50, CT50) was defined as the toxin concentration that resulted in 50% reduction of the absorbance observed with untreated control.

### 2.8. Preparation of Bacterial Culture Supernatants Containing Native CPB2

Since CPB2 is a secreted protein [4], culture supernatants of the* C. perfringens* C58-1 and C59-44 isolates were collected and used for examining the presence of CPB2 protein. For this purpose,* C. perfringens* was grown anaerobically overnight at 37°C in Schaedler anaerobe broth (Oxoid Limited, UK) and for CPB2 production [20]. The culture supernatant was collected by centrifugation at 12,100 ×g for 20 min at 4°C. The supernatant was then filtered using a 0.22 *μ*m Nalgene Bottletop Filter (Merck Millipore Company, Germany). Samples were used for SDS-PAGE and Western blot analysis of secreted proteins and stored at −20°C for future use.

### 2.9. Characterization of McAbs by an Immunoblotting Assay

The rCPB2 toxin protein or the culture supernatant and cell lysis of* C. perfringens* C58-1 (type B) and C59-44 (type C) strains were separated by SDS-PAGE on 12% gels. The resolved proteins were transferred to a PVDF membrane using a Bio-Rad mini-transfer apparatus (Bio-Rad, Hercules, USA). After blocking with 5% BSA in TBS (20 mM Tris-HCl, 137 mM NaCl, pH 7.6) for 1 h at room temperature, the membrane was first incubated with each of McAbs derived from distinct hybridoma cell lines. The ascites containing McAbs were diluted in TBST (20 mM Tris-HCl, 137 mM NaCl, 0.1%Tween-20, pH 7.6). After extensive washing, the membrane was incubated with secondary IRDye 800CW goat anti-mouse (LI-COR). Immune complexes were detected using the Odyssey dual-infrared fluorescence system.

### 2.10. Immunofluorescent Staining

The NCM460 cells cultured in collagen-coated cover slides were treated with rCPB2 toxin for different times (5 min, 30 min, 2 h, 4 h, 6 h, and 12 h) for evaluating the binding of rCPB2 to cell membranes. The rCPB2-treated cells were fixed with 4% paraformaldehyde in PBS at room temperature for 15 min, washed in PBS for 3 × 5 min, and permeabilized with 0.3% Triton X-100 for 10 min at room temperature. Nonspecific antibody binding was blocked using 5% normal donkey serum in PBS for 1 h at room temperature, after which primary antibodies against CPB2 toxin were applied at a 1 : 100 dilution in PBS and incubated at 4°C overnight. Primary antibody binding was detected using the FITC-labeled donkey-anti-mouse IgG secondary antibody (1 : 500) (Thermo, Rockford, USA). After extensive washing, cell membranes were mounted by Annexin A2 Polyclonal Antibody (1 : 200) (SanYing Biotechnology, China) as primary antibody, and Rhodamine- (TRITC-) conjugated goat anti-rabbit IgG as secondary antibody (1 : 250) (SanYing Biotechnology, China). After extensively washing, the slides were mounted for fluorescence in Vectashield Mounting Medium with DAPI (Thermo, Rockford, USA). Images were acquired using a Leica TCS SP2 A0BS Confocal System and processed on Leica Confocal Software v.2.6.1 (Leica, Germany).

### 2.11. Enzyme Linked Immunosorbent Assay (ELISA)

96-well plates were coated with recombinant CPB2 (3 *μ*g/mL) protein in 0.1 mol/L sodium carbonate buffer overnight at 4°C. The plates were then blocked with 5% BSA in PBS for 2 h at room temperature to prevent nonspecific binding. After plates were washed with 0.05% Tween in PBS, 2-fold series of dilution of McAb ascites to CPB2 were added to wells, and the plates were incubated at 37°C for 2 h. Followed by being washed with 0.05% Tween in PBS, the diluted goat anti-mouse IgG conjugated with horseradish peroxidase (from Zhongshan Biological Inc., Beijing, China) was added to the immunoplates and incubated for 1 h at room temperature. Color was developed by adding TMB (3,3,5,5′-tetramethylbenzidine) substrate solution containing 0.03% H_2_O_2_. Absorbance was read at 450 nm after quenching the wells with 50 *μ*L of 2 mol/L H_2_SO_4_.

### 2.12. *In Vitro* Neutralization Assay

The capacity of monoclonal antibodies to neutralize the cytotoxicity of rCPB2 toxin was determined by an* in vitro* neutralization assay. Briefly, 2x CT50 (30 *μ*g/mL) of rCPB2 toxin was mixed with 200 *μ*L of 50x diluted ascites of monoclonal antibodies. The mixture was incubated for 2 h at 37°C. The mixtures of McAb and rCPB2 toxin proteins were then applied into the cells and incubated at 37°C for additional 12 h. The control wells were treated with an equal volume of RPMI-1640 supplemented with 10% FCS. The RPMI-1640 supplemented with 10% FCS was used as solvent for dilution. The cell viability of the NCM460 cells was measured by MTT assay.

### 2.13. Statistical Analysis

All data collected in this study were obtained from at least three independent experiments for each condition. SPSS18.0 analysis software (PC version, SPSS Inc., Chicago, IL, USA) was used for the statistical analysis. Statistical evaluation of the data was performed by a *t*-test for comparison of differences between two groups. A difference was considered to be a statistical difference at a *p* value of <0.05. Data was presented as the mean ± standard deviations (SD).

## 3. Results

### 3.1. Expression and Purification of Recombinant CPB2 Toxin Protein

By employing general DNA cloning protocols and a PCR strategy, the CPB2-toxin encoding gene (*cpb2*) was in-frame cloned into the pTIG-Trx bacterial backbone plasmid to generate a vector expressing* cpb2-his* tag fusion gene (Figure 1(a)) [16].* E. coli* BL21 (DE3) cells harboring pTIG-Trx-CPB2 plasmid were induced for transgenic expression in the presence of IPTG. SDS-PAGE analysis showed an expression of recombinant His-tagged CPB2 protein with expected MW of ~28 kDa in the soluble fraction of bacterial cells, which was able to bind to Ni-NTA column and be purified by HPLC AKTA PURIFIER PH/C10 system with a gradient imidazole (lanes 5–7 in Figure 1(b)). The peak of purified protein of interest was observed in the imidazole-eluted fraction at concentration of 75 mM (Figure 1(b)). Immunoblotting analysis further revealed that the purified proteins were specifically reacted with 1E23 antibody (1 : 800) against CPB2 (Figure 1(c)). Notably, detectable native CPB2 protein was observed in the culture supernatant of C59-44 isolate (lane 9 in Figure 1(c)) but not in the cell lysate (lane 8 in Figure 1(c)), as determined by the immunoblotting assay (Figure 1(c)). These results suggested that the purity and integrity of rCPB2 generated by this approach could be used for investigation of biological functions of CPB.

### 3.2. Cytotoxicity of rCPB2 in NCM460 Intestinal Epithelial Cells

**Figure 2:**
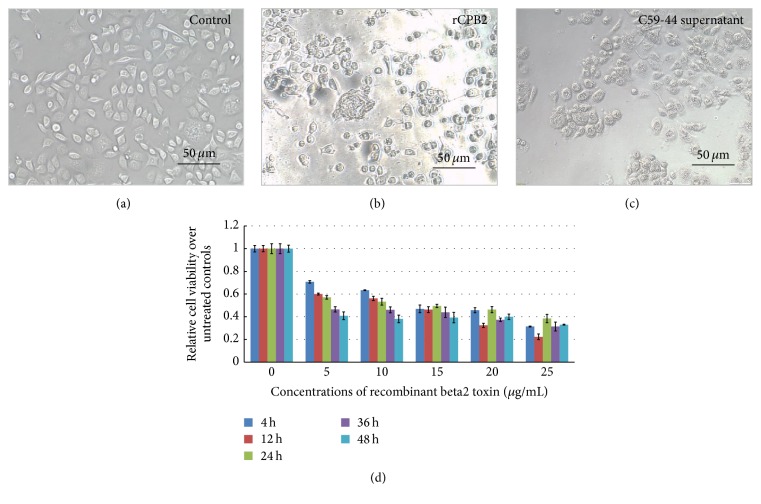
The cytotoxicity of rCPB2 toxin in human colon NCM460 cells. The cytotoxicity of purified rCPB2 protein was ascertained by determining its concentration causing 50% cell death (cytotoxicity 50, CT50) in a given time point in terms of an MTT assay. (a) A representative image (×400) of control NCM460 showed normal morphology of cells. (b) A representative image of NCM460 cells treated with rCPB2 at 20 *μ*g/mL for 12 h exhibited a morphological change of cell death. (c) A representative image of NCM460 was treated for 12 h with supernatant of* C. perfringens* C59-44 strain. (d) The viability of NCM460 cells treated with indicated concentrations of rCPB2 for indicated times determined by an MTT assay. The data was expressed as the mean ± SD of fold changes of viable cells against the control untreated cells from three triplicated independent experiments. Bars in (a) and (b): 50 *μ*m.

The cytotoxic activity of purified rCPB2 toxin was assessed in terms of its effects on NCM460 cells by an MTT assay. Morphological changes were observed in cells exposed to 20 *μ*g/mL of rCPB2 toxin (Figure 2(b)), including rounding up of cells and ultimately leading to cell death, as compared with the untreated control cells (Figure 2(a)). Of interest, the degree of 20 *μ*g/mL of rCPB-induced cytotoxicity was comparable to the culture supernatant of* C. perfringens* C59-44 strain, as determined by morphological observation. This result indicates that the rCPB2 toxin has a biological activity of cytotoxicity* in vitro*. MTT assay further revealed a dose-dependent cytotoxicity of rCPB2 on NCM460 cells, but a time-dependent cytotoxicity of this recombinant toxin on cells was only observed at a dosage less than 15 *μ*g/mL (Figure 2(d)). The cytotoxic activity was observed at a concentration as low as 5.0 *μ*g/mL, and the concentration causing 50% cell death (CT50) was determined at 15 *μ*g/mL (Figure 2(d)).

### 3.3. rCPB2 Induces Cell Apoptosis in NCM460 Cells

**Figure 3:**
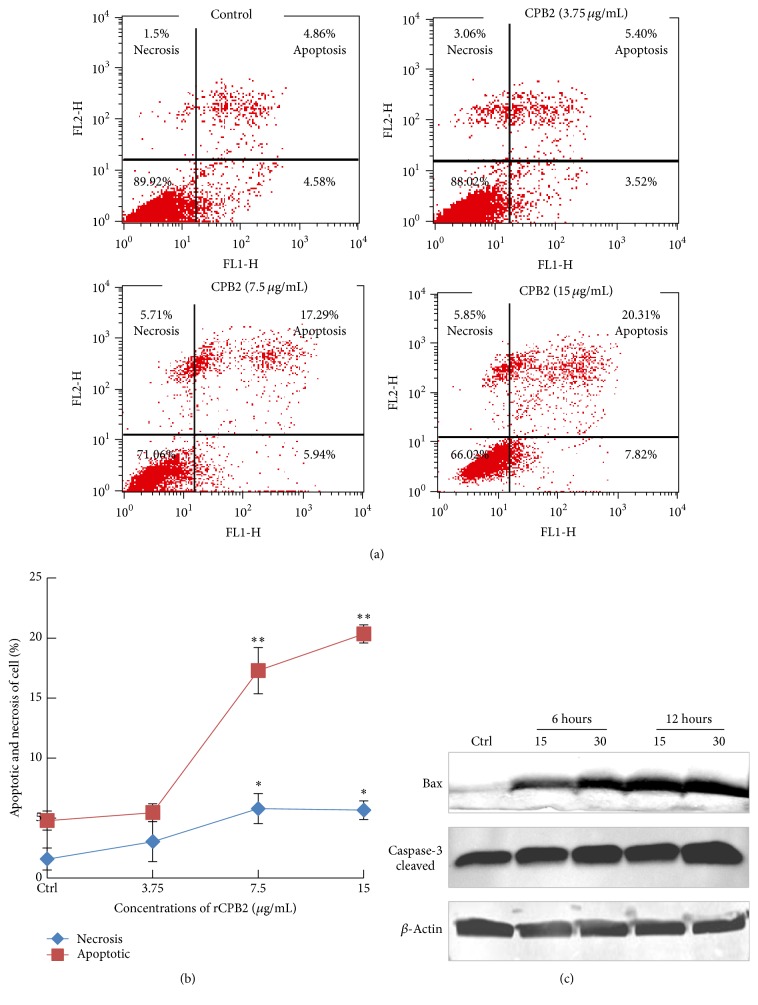
rCPB2 toxin induces apoptosis in NCM460 cells. The NCM460 cells were treated with indicated concentrations of rCPB2, and the cell death was analyzed by a cytometric assay. (a) Representative dot plots of three independent experiments of flow cytometry analysis showed an increased fraction of apoptotic NCM460 cells treated with indicated concentrations of rCPB2 for 12 h. (b) The quantitative analysis showed a dose-dependent cell apoptosis induced by rCPB2; the rCPB2 exhibited an ability to induce cell apoptotic death rather than necrosis. (c) Immunoblotting assay of proapoptotic protein caspase-3 and Bax showed potential of rCPB2-induced NCM460 cells. Data represented the mean ± SD from three independent experiments in (b) compared to a naive control, ^*∗*^ *p* < 0.05; ^*∗∗*^ *p* < 0.01.

Next we sought to examine a potential mechanism of cell death induced by rCPB2. Flow cytometric analysis showed more frequency of apoptotic cells when they were exposed to rCPB2, in comparison with untreated cells (Figure 3(a)) In addition, the rCPB2-induced cell apoptosis was dose-dependent (Figure 3(b)). Intriguingly, rCPB2 exhibited potential to induce an apoptotic cell death rather than a necrotic cell death, as determined by the cytotoxicity assay (Figure 2). In this regard, rCPB2 induced up to 20% cells with apoptotic cell death relative to about 5% of cells with necrotic death when NCM460 cells were cultured in the presence of a dose of CT50 (Figure 3(b)). Mechanistically, immunoblotting analysis showed more abundant proapoptotic proteins, including caspase-3 and Bax in cells treated with rCPB2 (Figure 3(c)). This data implies an involvement of caspase-dependent apoptotic pathway in CPB2-mediated cell apoptosis. However, the precise mechanism by which CPB2 toxin induced cytotoxicity needs further investigation.

### 3.4. Characterizations of Monoclonal Antibodies against CPB2

**Figure 4:**
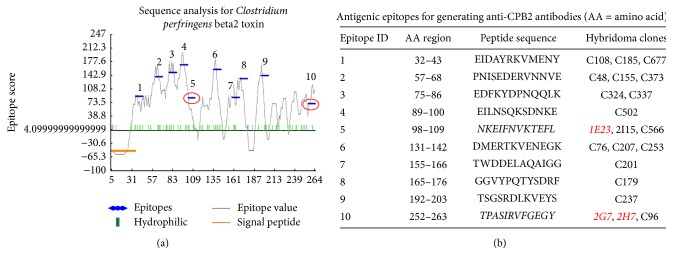
Generation of hybridoma cell lines producing antibodies against CPB. Ten predicted antigenic epitopes were used for generation and screening of antibodies against CPB2 using a SEAL technology (Surface Epitope Antibody Library) developed by Abmart (Shanghai, China). (a) A graph showed an overall structure of protein of interest and the 10 epitopes selected peptide for immunization to generate hybridoma cell lines producing specific antibodies against CPB2. The value of *X*-axis indicated locations of amino acids (AAs) from N-terminal to C-terminal of CPB2 protein; the value of *Y*-axis indicated scores of algorithm, a high score represented an epitope with high possibility. The ten selected epitopes (1–10) were highlighted with blue on the graph. The epitopes 5 and 10 were circled in red, and antibodies to epitopes 5 and 10 were further identified to react with rCPB2 in this study. (b) The IDs, AA regions, AA sequence, and hybridoma cell clones of respective epitopes examined in this study. The peptide sequences in italic were able to generate cell clones producing antibodies that were identified to react with rCPB2 (clones 1E23, 2G7, and 2H7, labeled in red and italic).

**Figure 5:**
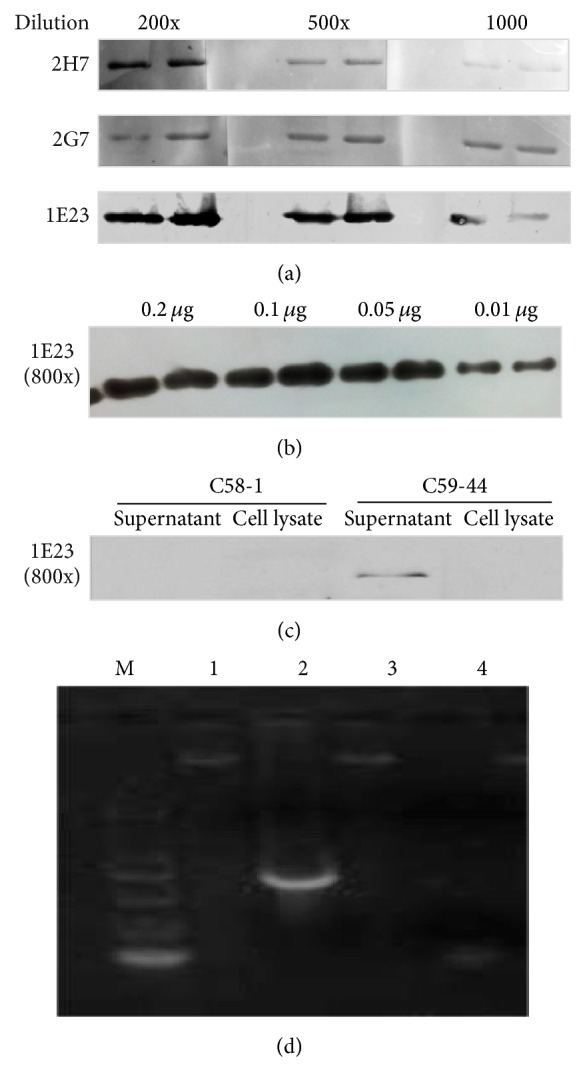
Characterization of monoclonal antibodies to rCPB2 by an immunoblotting assay. Monoclonal antibodies (McAbs) produced by hybridoma cell clones listed in Figure 4(b) were tested for their cross-reactions with rCPB2 and native CPB2 in terms of an immunoblotting assay. (a) Images of immunoblots of monoclonal antibodies produced by 2H7, 2G7, and 1E23 hybridoma cell clones against 0.1 *μ*g of rCPB2 with indicated dilutions of ascites generated from Balb/C mice. (b) The sensitivity of immunoblotting assay in detection of rCPB2 using McAb 1E23 at a dilution of 800. (c) The sensitivity of immunoblotting assay in detection of native CPB2 in the culture supernatants and cell lysates of* C. perfringens* type B (C58-1) strain (left panel) and type C (C59-44) strain (right panel) McAb 1E23 at a dilution of 800. (d) A representative image of ethidium bromide (EB) stained agarose gel of genomic DNA and PCR product of cpb2 gene fragment. Lines 1 and 2 were respective C59-44 genomic DNA and PCR fragment of cpb2 genes and lines 3 and 4 were C58-1 genomic DNA and PCR product, respectively. The immunoblotting results showed that McAb 1E23 could react with both recombinant CPB protein and native CPB2 produced by* Clostridium perfringens* type C.

**Figure 6 fig6:**
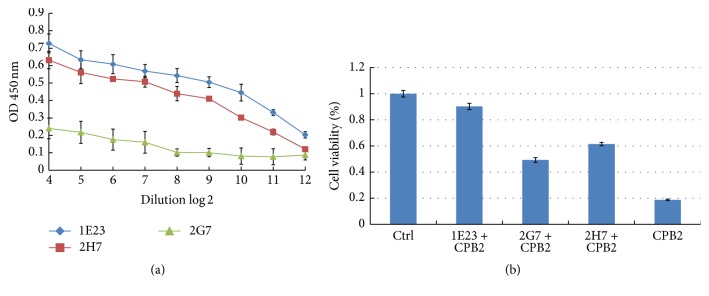
Characterizations of McAbs to CPB2 by an ELISA and abilities to neutralize rCPB2-induced cytotoxicity* in vitro*. (a) Titration of ascites of McAbs to CPB2 determined by an ELISA. An ELISA plate was coated with rCPB2 at concentration of 3.0 *μ*g/mL; ascites of McAbs were diluted by 2-fold series dilution and incubated with above coated antigens; a reaction of antigen-antibody was detected by an ELISA. The result indicated that McAbs 1E23, 2G7, and 2H7 could be used for detecting CPB by an ELISA. (b) A capacity of McAbs to neutralize the toxicity of rCPB2 toxin* in vitro*. A 2x LD50 (30 *μ*g/mL) of rCPB2 toxin was mixed with 200 *μ*L of 50x diluted McAbs and incubated at 37°C for 2 h; the mixture was then applied to NCM460 cells incubated at 37°C for additional 12 h. The cell viability was measured by an MTT assay. The result showed abilities of tested McAbs to neutralize the cytotoxicity of rCPB2* in vitro*. Among these McAbs, 1E23 displayed the most potential to neutralize the cytotoxicity of rCPB at a dose of 30 *μ*g/mL.

**Figure 7:**
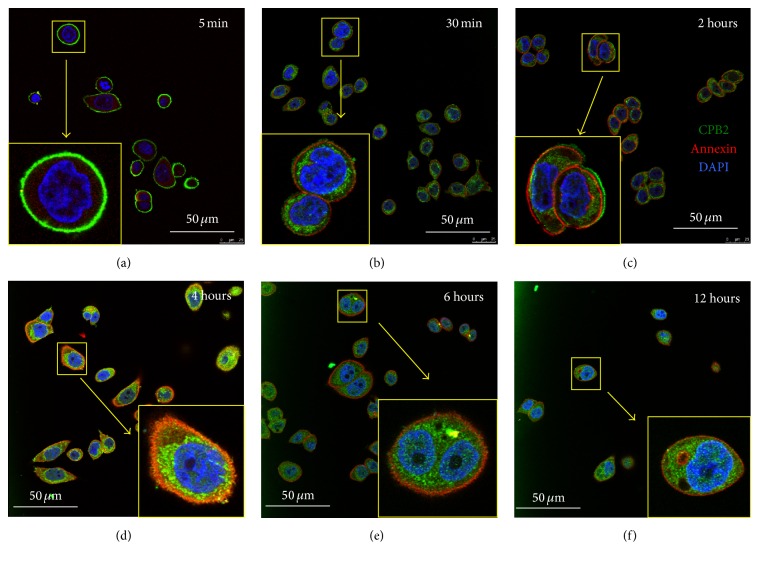
Ability of rCPB2 binding to NCM460 cells characterized by an immunofluorescent assay. NCM460 cells were incubated with rCBP2 at concentration of 15 *μ*g/mL for indicated times. The cells were then used for accessing rCPB2 protein and cell membrane with McAb 1E23 to CPB2 (1 : 100) and rabbit polyclonal antibody to Annexin A2 (1 : 200), respectively. FITC-labeled donkey anti-mouse IgG (1 : 500) and Rhodamine- (TRITC-) conjugated goat anti-rabbit IgG (1 : 250) were used as secondary antibodies for visualizing respective proteins of interest. DAPI was used for nuclear staining. (a–f) Representative images of immunofluorescent staining for cells incubated with rCPB2 at indicated times of 5 min (a), 30 min (b), 2 h (c), 4 h (d), 6 h (e), and 12 h (f). The results showed that rCPB2 was able to bind to the membrane of NCM460 cell and translocate into the cytoplasm of cells. Insets were the enlarged boxed regions of respective images in (a–f). Bars in (a–f): 50 *μ*m.

In order to further explore biological roles of CPB2 as well as provide a useful tool for detection of CPB2 toxin in clinical settings and investigation of functional mechanisms of this bacterial toxin, McAbs to CPB2 were produced by hybridoma cell lines generated from mice immunized with a mixture of 10 antigenic epitopes that integrated Abmart antigen system (Figures 4(a) and 4(b)). A total of 21 hybridoma cell lines were generated; among them, clones 1E23, 2G7, and 2H7 were identified to cross-react with both native CPB2 and rCPB2 by several immunological assays (Figures 5 [Fig fig6]–7). McAb 1E23 was derived from CPB2 epitope number 5 which spans AA region of 98–109 (NKEIFNVKTEFL), while McAbs 2G7 and 2H7 were generated from mice immunized with epitope number 10 which encompasses AA sequence of 252–263 (TPASIRVFGEGY) (Figure 4). Of note, these McAbs could detect both rCPB2 and native CPB2 by an immunoblotting assay using purified rCPB2 and supernatants of* C. perfringens* C59-44 isolate (Figure 5). All three selected McAbs showed ability to cross-react with rCPB2 in the immunoblotting assay; among them, McAb 1E23 exhibited the best immunological reactivity in this assay (Figure 5(a)), which could detect 10 ng (0.01 *μ*g) of rCPB2 (Figure 5(b)) and thus was used for further analysis in this report. The 1E23 antibody was also capable of detecting native CPB2 in the supernatant of* C. perfringens* C59-44 strain but not C58-1 strain by immunoblotting assay (Figure 5(c)). Consistently, the* cpb2* gene was only amplified in* C. perfringens* C59-44 strain but not in C58-1 strain by PCR (Figure 5(d)), suggesting that the undetectable CPB2 in* C. perfringens* C58-1 strain was due to the lack of* cpb2* gene. Similarly, these antibodies were also able to cross-react with rCPB2 in an ELISA, in which McAbs 1E23 and 2H7 displayed a greater affinity with a titration of ascites up to 4000, relative to McAb 2G7 by this assay (Figure 6(a)). More importantly, all the three tested McAbs exhibited potential to neutralize the cytotoxicity of rCPB2 on NCM460 cells (Figure 6(b)). For* in vitro* neutralization assay, 2x CT50 of rCPB2 toxin protein (30 *μ*g/ml) was preincubated with 50x diluted McAb ascites at 37°C for 2 h, and then the mixture was applied on NCM460 cells. Results from the MTT assay demonstrated a different extent of protection of cells from cytotoxicity of rCPB2 among these three antibodies; among them 1E23 was the most powerful in neutralizing rCPB2 cytotoxicity* in vitro*, which could result in an up to 90% NCM460 cell viability against toxicity of 2x CT50 of rCPB2 as compared with the untreated control cells. Viabilities of cells exposed to rCBP2/2G7, rCPB2/2H7, and rCPB2 alone were 44%, 61%, and 19% in comparison with the untreated cells, respectively (Figure 6(b)).

### 3.5. rCPB2 Binds to Membrane and Dynamically Translocates into Cytoplasm of NCM460 Cells

In an attempt to understand the underlying mechanism of cytotoxicity of CPB2* in vitro*, the capacity of rCPB2 to bind to and translocate into cells was determined by an immunofluorescent (IF) assay using McAbs 1E23, 2G7, and 2H7. The IF staining revealed a specific fluorescent staining of rCPB2 in cells probed with McAb 1E23 but not with McAbs 2G7 and 2H7 (Figure 7 and data not shown). Interestingly, a dynamic translocation of rCPB2 toxin protein into the NCM460 cells was observed with the lapse of time from 5 min to 12 h, as determined by the IF assay using antibody 1E23 (Figures 7(a)–7(f)). At 5 min of incubation, the rCPB2 protein was only observed in the outer membrane of cells (Figure 7(a)). At 30 min of incubation, the recombinant toxin could be determined in both membrane and cytoplasm of cells (Figure 7(b)) and more abundant rCPB2 protein was transported into cytoplasm at 2 h after the incubation (Figure 7(c)), which led to an increased intensity of staining for cell membrane marker Annexin A1 but a diminished rCPB2 staining over the time. Of note, the majority of rCPB2 protein was found in the cytoplasm but few proteins were still bound to cell membrane at 4 h after incubation (Figure 7(d)). However, rCPB2 toxin only was observed in cytoplasm at incubations of 6 h (Figure 7(e)) and 12 h (Figure 7(f)). These observations clearly suggested that the rCPB2 toxin protein could bind to the cell membrane and dynamically translocate into cytoplasm with time. This result further indicated a biological activity of purified rCPB2 and implied the usefulness of McAb 1E23 in studying the functional mechanism of cytotoxicity of CPB2.

## 4. Discussion

Necrotic enteritis (NE) is a worldwide disease caused by infection of* C. perfringens*, a ubiquitous anaerobic bacterium that is readily found in soil, dust, feces, feed, poultry litter, and intestinal contents [21–23]. Pathologically, varied toxins produced by* C. perfringens* are main virulent factors of this species of pathogen; therefore, a better understanding of biological activities and underlying mechanisms of their cytotoxicity will provide an insight into the pathogenesis of* C. perfringens* infection. However, these studies have been significantly hampered, partially owing to the difficulty in purification of* C. perfringens* toxins and the lack of their specific antibodies. In the present report, we generated and characterized a recombinant His-tagged* C. perfringens* beta2 (rCPB2) toxin and McAbs against CPB2* in vitro*. Our results showed that rCPB2 purified from* E. coli* had limited extent of cytotoxicity on NCM460 human intestinal epithelial cells (Figures 1 and 2), on which rCPB2 induced more frequency of apoptotic cell death than the necrotic main cell death, at least in part through an apoptotic pathway involved in caspase-3 signaling (Figure 3). In addition, three McAbs produced by hybridoma cells 1E23, 2G7, and 2H7 were characterized to be able to cross-react with both rCPB2 and native CPB2 by assays of immunoblotting, ELISA, and immunofluorescence (Figures 4 and 5). Importantly, these antibodies exhibited a capacity to neutralize the cytotoxicity of rCPB2 on NCM460 cells (Figure 6). More notably, the immunofluorescent staining further revealed an ability of rCPB2 to bind to cell membrane prior to being dynamically transported into cytoplasm (Figure 7).

Mounting evidence has revealed that a purified bacterial toxin can elicit characteristic symptoms of the diseases caused by infection of the bacteria producing this toxin in experimental animals, a direct implication of the toxin in pathogenesis. Therefore, vaccination against the toxin is the only preventive measure against this disease. Despite the fact that native toxin(s) can be produced by most bacteria in culture, it is difficult to be purified; production of toxins through recombinant DNA technology is thus an advantage as it bypasses the need to culture the pathogen for purification using conventional methods. Furthermore, for development of an effective vaccine, it is important for a recombinant protein to retain all properties of its native toxin [20]. In this regard, the purified His-tagged rCPB2 toxin protein generated from an* E. coli* expressing system in this report retains integrity, cytotoxicity, and capacity to bind to cell membrane of a native CPB2 [4]. These biological activities of rCPB2 were in line with those found in purified native CPB2 derived from a porcine* C. perfringens* strain [4] and human* C. perfringens* strain [9], in which CPB2 showed a cytotoxic activity on human intestinal cells (I407) and human Caco-2 cells, respectively. However, a minimal concentration of cytotoxicity for native CPB2 has been reported in great variations, with a range from 0.1 *μ*g/mL to 20.0 *μ*g/mL [4]. Such a variation in doses required for cytotoxicity was due to the instability, and/or it might be a cell context-dependent manner of cytotoxic mechanism of this type of toxin [4]. In a previous work by Gibert et al., the authors found that the native of CPB2 failed to significantly disrupt the cytoskeleton of human intestinal (I407) cells, which was unlikely for other toxins produced by* C. perfringens* species [4]. This notion was supported by a recent study on the cytotoxicity of CPB2 toxin of human and porcine* cpb2*-harbouring* C. perfringens* [24]. In this report, the authors demonstrated that there was no significant role for CPB2 in the cytotoxicity of human Caco-2 cells and porcine IPI-21 cells induced by human and porcine* cpb2*-harbouring* C. perfringens* [24]. Such a discrepancy in cytotoxicity of CPB2 was also previously reported in a study on the association of CPB2 and* C. perfringens* type A isolates carrying a plasmid enterotoxin gene in human gastrointestinal disease [9]. In this study, Fisher et al. demonstrated that CPB2 could be an accessory toxin in* C. perfringens* enterotoxin- (CPE-) associated AAD/SD. Molecularly, two different CPB2 variants (named CPB2h1 and CPB2h2) could be produced by AAD/SD isolates, which were dependent on whether IS1470-like or IS1151 sequences were present downstream of their* cpe* gene. Cytotoxically, CPB2h1 showed approximately 10-fold more cytotoxicity for CaCo-2 cells relative to CPB2h2, despite the fact that both purified CPB2h1 and CPB2h2 could induce CaCo-2 cell damage after the confluent monolayer cell cultures were exposed to the purified toxins at a concentration of 10 *μ*g/mL for 5.5 h [9]. In line with these findings, we also showed that the purified rCPB2 only exhibited a low cytotoxicity with an up to 15 *μ*g/mL of CT50 on NCM460 cells in this study (Figure 2).

With respect to antibodies against CPB2 generated in this study, a mixture of peptides of 10 optimized potential epitopes that were predicted by a software developed by Abmart were integrated into Abmart in-house antigen system which was used for immunization of mice for generation of hybridoma cells producing McAbs (Figure 4). Three hybridoma cell lines that produce respective McAbs 1E23, 2G7, and 2H7 were screened to be able to immunologically react with both rCPB2 and native CPB2 as determined by immunoblotting assay, ELISA, and immunofluorescent staining (Figures 5 [Fig fig6]–7). In particular, McAb 1E23 displayed the broadest and most specific immunological reaction to rCPB2 and native CPB2 among these three McAbs in all tested assays (Figures 5 [Fig fig6]–7). These antibodies will provide useful tools for investigating pathogenic mechanisms of CPB2 and developing assays for detection as well as neutralizing this toxin in clinical settings. Indeed, all three screened McAbs showed a varied extent capacity to neutralize the cytotoxicity on NCM460 cells, and McAb 1E23 was the most powerful in neutralizing cytotoxicity (Figure 6). Mechanistically, immunofluorescent analysis clearly demonstrated that rCPB2 was first bound to cell membrane and then translocated into cytoplasm dynamically* in vitro* (Figure 7). Flow cytometric analysis showed that the majority of cell death induced by rCPB2 was cell apoptosis rather than a necrotic death on NCM460 cells (Figure 3). Molecular analysis further revealed that a caspase-3 signaling was involved in the rCPB2-induced cell apoptosis (Figure 3). These findings provide important information on mechanisms of biological functions of CPB2 for the first time, an indicative of usefulness of the rCPB2 toxin protein and antibodies.

## 5. Conclusion

Collectively, in the present report, we first generated and purified a His-tagged recombinant beta2 toxin of* C. perfringens* (rCPB2) and produced monoclonal antibodies (McAbs) against CPB2. The purified rCPB2 toxin expressed in* E. coli* retained the integrity and some extent of cytotoxicity of CPB2. Three screened McAbs to CPB2 could immunologically react with both rCPB2 and native CPB2 by immunoblotting assay, ELISA, immunofluorescent staining, and neutralization analysis. Mechanistically, rCPB2 was able to first bind to cell membrane and dynamically translocate into cytoplasm for its cytotoxic activity. Flow cytometric analysis further revealed that rCPB2 could induce apoptotic cell death rather than necrotic death in NCM460 cells and caspase-3 signaling was involved in this apoptotic process. However, the precise mechanisms of the cytotoxicity and pathogenesis need to be further investigated by using the rCPB2 toxin protein and antibodies produced in this study.

## Abbreviations

AAs: Amino acids
AAD/SD: Antibiotic-associated/sporadic diarrhea
Bax: Bcl-associated X protein
BSA: Bovine serum albumin
*C. perfringens*: * Clostridium perfringens*
Caspase: Cysteinyl aspartate specific proteinase
CT50: Cytotoxicity 50, which is a concentration of toxin protein that resulted in 50% cell death in this study
DAPI: 4′,6-Diamidino-2-phenylindole
ECL: Electrochemiluminescence
ELISA: Enzyme linked immunosorbent assay
HRP: Horseradish peroxidase
IF: Immunofluorescence
IPTG: Isopropyl *β*-D-1-thiogalactopyranoside
McAbs: Monoclonal antibodies
MTT: 3-(4,5-Dimethylthiazol-2-yl)-2,5-diphenyltetrazolium bromide
Ni-NTA: Nickel-nitrilotriacetic acid
PVDF: Polyvinylidene fluoride
rCPB2: Recombinant* C. perfringens* beta2 toxin
SDS-PAGE: Sodium dodecyl sulphate polyacrylamide gel
SEAL: Surface epitope antibody library
TMB: 3,3′,5,5′-Tetramethylbenzidine.
